# FRONTIER1 multiple ascending dose extension: a safety, tolerability, pharmacokinetics, and pharmacodynamics study of Mim8 in people with hemophilia A

**DOI:** 10.1016/j.rpth.2025.103207

**Published:** 2025-10-08

**Authors:** Pratima Chowdary, Steven R. Lentz, Lidia Gil, Francisco J. López-Jaime, Jerzy Windyga, Wan Hui Ong Clausen, Peter Nørkjær Laursen, Johnny Mahlangu

**Affiliations:** 1 Katharine Dormandy Haemophilia and Thrombosis Centre, Royal Free Hospital, Department of Haematology, University College London, London, UK; 2 Department of Internal Medicine, University of Iowa, Iowa City, Iowa, USA; 3 Department of Hematology and Bone Marrow Transplantation, Poznań University of Medical Sciences, Poznań, Poland; 4 Department of Hemostasis and Thrombosis, Hematology Service, Hospital Universitario Regional de Málaga, Málaga, Spain; 5 Department of Hemostasis Disorders and Internal Medicine, Laboratory of Hemostasis and Metabolic Diseases, Institute of Hematology and Transfusion Medicine, Warsaw, Poland; 6 Novo Nordisk A/S, Søborg, Denmark; 7 Department of Molecular Medicine and Haematology, University of the Witwatersrand, National Health Laboratory Service, and Charlotte Maxeke Johannesburg Academic Hospital, Johannesburg, South Africa

**Keywords:** Bispecific antibody, factor VIIIa, hemophilia A, pharmacokinetics, safety

## Abstract

**Background:**

Mim8 (denecimig) is a next-generation, factor VIIIa mimetic, human bispecific antibody for hemophilia A. Mim8 was well tolerated in the phase 1/2 FRONTIER1 (NCT04204408) dose-escalation part.

**Objectives:**

Report Mim8 long-term safety in the FRONTIER1 multiple ascending dose (MAD) extension phase.

**Methods:**

People (≥12 years) with severe hemophilia A with/without inhibitors who completed the 12-week FRONTIER1 MAD main phase enrolled in the extension and continued Mim8 once weekly or once every 4 weeks for up to 148 weeks; patients could switch doses or transition to maintenance doses designed to achieve target exposure in phase 3 trials. Primary endpoint: treatment-emergent adverse events (TEAEs). Secondary endpoints: injection-site reactions, anti–Mim8 antibodies.

**Results:**

Forty-one patients entered the extension; *n* = 39 switched to Mim8 maintenance doses administered using tiered dosing by body weight (30–<60 kg or ≥60 kg): *n* = 31 received once-weekly dosing (6 or 11 mg); *n* = 9 received once-every 4-weeks dosing (30 or 46 mg); *n* = 1 patient switched between doses. Total exposure: 69.5 patient-years. Thirty-five patients experienced 174 TEAEs. Seven serious TEAEs occurred (none Mim8-related). TEAE rate, type, and severity were not dose-dependent. Of 3128 injections, 6 (0.2%) injection-site reactions were recorded in 6 (14.6%) patients. There was no evidence of anti–Mim8 antibodies. Mim8 exposure and peak thrombin levels remained consistent across cohorts and maintenance doses. No dose-dependent changes in D-dimer, prothrombin fragment 1 + 2, or fibrinogen levels were observed. Most patients experienced no bleeds during maintenance dosing.

**Conclusion:**

Mim8 was well tolerated, with no safety concerns nor anti–Mim8 antibodies during the FRONTIER1 MAD extension, supporting phase 3 development of Mim8 once weekly and once every 4 weeks.

## Essentials

• Mim8 is a FVIIIa mimetic developed for hemophilia A with or without inhibitors (87/98 characters including spaces).
• The phase 2 FRONTIER1 study extension phase assessed long-term safety of Mim8 up to 148 weeks (96/98 characters including spaces).
• Mim8 exposure and thrombin generation were dose-dependent (59/98 characters including spaces).
• Mim8 was well tolerated, with no substantial safety concerns or anti–Mim8 antibodies observed (95/98 characters including spaces).

## Graphical abstract

**Figure:**
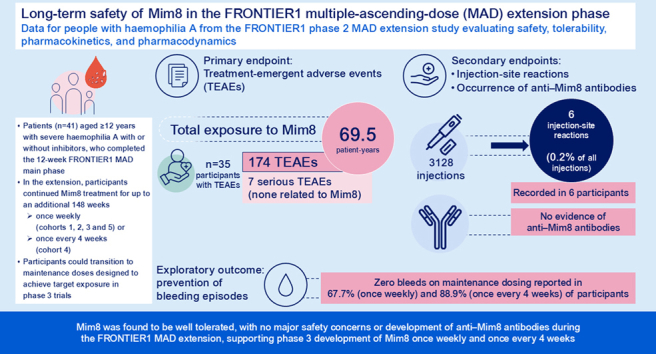

## 1 Introduction

Hemophilia A is an X-linked congenital bleeding disorder, with a prevalence of approximately 1:5000 males, characterized by a deficiency of factor (F)VIII [1]. Insufficient thrombin generation, impaired hemostasis, prolonged bleeds, and re-bleeds in people with hemophilia can contribute to long-term morbidity and impaired quality of life [2]. The current standard-of-care treatment for severe and moderate hemophilia A is prophylactic FVIII replacement or activated FVIII (FVIIIa) mimetics. Unmet needs in hemophilia include mental health burden (eg, anxiety that treatment does not confer protection) [3], high treatment burden (storage, preparation, and frequent infusion and painful administration) [4], and safety risks (eg, thrombotic risk [5] and injection-site reactions [6]).

Nonreplacement therapies aim to improve treatment efficacy, maintain safety, and reduce treatment burden through subcutaneous administration and reduced treatment frequency [7,8]. These therapies can be used in patients who have developed inhibitors against factor replacement and in patients without inhibitors [7,8]. Among nonreplacement therapies are FVIIIa mimetics [9], of which emicizumab was the first to be approved for prophylaxis for people with hemophilia A with or without inhibitors [6,10].

Mim8 (denecimig) is a next-generation, optimized, FVIIIa mimetic, human bispecific immunoglobulin G4 antibody in clinical development for hemophilia A with or without FVIII inhibitors. Mim8 binds activated coagulation factor IX (FIXa) and factor X (FX), mimicking the cofactor function of FVIIIa and positioning them for the enzymatic conversion of FX to activated FX by FIXa. Mim8 has been optimized for low binding affinity to FX and FIXa and high cofactor activity [11], reducing the risk of nonspecific binding while providing efficient FX activation on the surface of activated platelets at the injury site. Mim8 has a long half-life (∼30 days), enabling reduced frequency of injections (once every week to once every month) [12].

FRONTIER1 (NCT04204408) was a phase 1/2 study that investigated the safety, tolerability, pharmacokinetics (PK), and pharmacodynamics (PD) of single ascending doses of Mim8 in healthy participants and multiple ascending doses (MAD) of Mim8 in people with hemophilia A, with or without FVIII inhibitors. Results showed that Mim8 was well tolerated, and no safety concerns were observed. Additionally, Mim8 demonstrated potential clinical benefit, with few participants experiencing treated bleeds beyond the cohort with the lowest Mim8 dosing [12,13]. *Ex vivo* data from FRONTIER1 measuring thrombin generation suggested that a 15-fold lower concentration of Mim8 compared with emicizumab was adequate to achieve similar thrombin peak height; [12] however, the clinical impact of this is unknown. Additionally, the PK and PD properties of Mim8 exhibited dose-proportional effects and were supported by the once weekly and once every 4-week dosing approaches [13].

Building on previous research, the FRONTIER1 MAD part extension phase study is the first to provide a comprehensive safety profile of Mim8 by examining extended exposure (up to 160 weeks) to both new and previously tested doses.

The FRONTIER1 MAD part extension phase assessed the long-term safety, PK, and PD of subcutaneous Mim8 in male adults and adolescents with hemophilia A with or without FVIII inhibitors. These data support ongoing phase 3 trials by characterizing the long-term safety of Mim8 and validating the selection of maintenance doses based on exposure and pharmacodynamic response.

## 2 Methods

### 2.1 Study conduct

FRONTIER1 (EudraCT: 2019-000465-20; NCT04204408; NN7769-4513) consisted of a first-in-human phase 1 single ascending doses part [12], and a phase 2 MAD part, which was a multinational open-label study in participants with severe hemophilia A (with or without FVIII inhibitors). The FRONTIER1 MAD part consisted of a 12-week main phase, during which patients received 1 of 5 cohort doses (previously published [13]), and a further extension phase of up to 148 weeks, during which the majority of patients transitioned to different Mim8 maintenance doses aimed to achieve Mim8 exposure as in phase 3 studies (Supplementary Figure 1).

The trial was conducted in accordance with the Declaration of Helsinki, the International Conference on Harmonization Good Clinical Practice Guideline, and the United States Food and Drug Administration 21 Code of Federal Regulations Parts 50, 56, and 312. Appropriate health authorities and an independent ethics committee/institutional review board approved the study. Written informed consent was taken before the start of any trial-related activities.

### 2.2 Eligibility criteria

Eligibility criteria for the main phase of the MAD part of the FRONTIER1 trial were described elsewhere [13]. Briefly, participants were males aged 12 to 64 years, with a body weight ≥30 kg, diagnosed with congenital hemophilia A with FVIII activity <1%, with or without inhibitors. A minimum of 5 bleeds in the 24 weeks before screening was required for participants treated on-demand.

Patients were only eligible for the MAD extension phase if they had completed the 12-week treatment period in the MAD main phase. Patients were excluded if they had a major surgery within 30 days prior to first trial product administration or a major surgery planned during the trial. Minor surgery was allowed (defined as any invasive operative procedure where only the skin, the mucous membranes, or superficial connective tissue is manipulated).

### 2.3 Study design

In the main phase of the MAD part, participants were enrolled in 1 of 5 ascending cohort doses of subcutaneous Mim8, as described previously [13]. Four cohorts (MAD cohorts 1, 2, 3, and 5) received once-weekly (QW) doses of Mim8, and 1 cohort (MAD cohort 4) received treatment once every 4 weeks (Q4W). Within each cohort, dosing was based on 2 body weight ranges: 30 to <60 kg and ≥60 kg, receiving the lower and higher doses, respectively. The dose levels administered were 1 mg and 1.2 mg (cohort 1); 2.4 mg and 3.8 mg (cohort 2); 11 mg and 15 mg (cohort 3); 41 mg and 60 mg (cohort 4); and 24 mg and 35 mg (cohort 5) (Supplementary Figure 1, Supplementary Table 1).

Mim8 was administered subcutaneously using a NovoPen 4 pen-injector device. Patients who completed 12-week treatment during the MAD main phase entered the MAD extension phase on the same QW or Q4W dosing regimens, referred to henceforth as the extension starting doses. Patients received Mim8 for up to 148 weeks (ie, up to week 160) in the extension phase, with an additional follow-up of 16 weeks after the last dose (ie, week 176). During the MAD extension phase, dose increases and switching between QW and Q4W dosing were permitted following a safety review. An additional safety visit 4 weeks after any dose or dosing frequency change was required. The frequency and dose could be further adjusted during this visit.

Participants were subsequently moved to 1 of 2 extension maintenance doses. These doses were selected to achieve mean average concentration of 5 to 7 μg/mL, which is the target level of Mim8 exposure used in the final dose setting for phase 3 pivotal trials (NCT05053139 and NCT05306418) [14]. Mim8 target exposure levels were based on data from the phase 1 PK trial (NCT0512747) and the main phase of FRONTIER1 MAD part, which were used to develop population PK and PK/PD models for thrombin generation and treated bleeds with tiered dosing [13,14]. Target exposure levels reflected doses used in FRONTIER1 MAD cohorts 3 (11/15 mg QW) and 4 (41/60 mg Q4W), which demonstrated similar Mim8 steady-state concentrations and overlapping thrombin generation profiles [13,14]. FRONTIER1 MAD cohort 5 (24/35 mg QW) did not result in substantially higher thrombin peak height compared with cohort 3, suggesting limited pharmacodynamic benefits of increased exposure at this level [13].

Most participants (all except 1) in the FRONTIER1 MAD extension phase switched from extension starting doses to extension maintenance doses, which corresponded to the doses selected for phase 3 evaluation: extension maintenance dose 1 (6 mg or 11 mg QW for weight ranges 30–<60 kg or ≥60 kg, respectively) or extension maintenance dose 2 (30 mg or 46 mg Q4W for weight ranges 30–<60 kg or ≥60 kg, respectively) (Supplementary Table 1). Participants were assigned to either dose based on their chosen frequency and investigator discretion.

### 2.4 Study endpoints

The primary endpoint of the MAD extension phase was the number of treatment-emergent adverse events (TEAEs) from week 12 up to week 176. Secondary endpoints were the number of injection-site reactions and occurrence of anti–Mim8 antibodies from week 12 up to week 176 (16 weeks after the last dose).

Additional safety laboratory assessments included changes from week 12 to week 176 in plasma levels of D-dimer, fibrinogen, and prothrombin fragment 1 + 2.

Blood samples were collected during patient visits to determine the plasma concentration and activity of Mim8. Individual profiles of Mim8 plasma concentration were measured using electrochemiluminescence immunoassay (Meso Scale Discovery assay for determination of NNC0365-3769 [Mim8] in human plasma). Anti–Mim8 antibodies were measured as described previously [13].

Blood samples for PD assessments were collected during patient visits. Thrombin generation was measured in platelet-poor plasma triggered with activated coagulation FXI on a Fluoroskan Ascent instrument (Thermo Fisher Scientific). Measurements were carried out with or without the addition of anti-FVIII antibodies to neutralize potential residual FVIII activity. Thrombin generation data, including thrombin peak height, were analyzed using Thrombinoscope software (Stago). Data are reported for the FRONTIER1 MAD part’s main and extension phases.

Data for bleeding episodes during the extension (week 12 to week 160) were recorded, comprising start/stop date and time, anatomical location, bleed cause, severity, and haemostatic medication.

### 2.5 Statistical analysis

Descriptive analyses were performed using SAS Enterprise Guide V8.3.

## 3 Results

### 3.1 Study participants

In total, 43 patients were enrolled in the FRONTIER1 MAD part main phase; a description of the demographics and baseline characteristics of these patients was published previously [13]. Two patients discontinued treatment, and 41 completed the MAD part main phase and enrolled in the MAD part extension phase. The mean age of participants during the MAD extension phase was 33.1 years (range, 14–64 years), including 6 adolescent participants (14.6%) (Table 1).

**Table 1:** Demographics and baseline characteristics of patients in the FRONTIER1 multiple ascending doses part extension phase.

Demographics and baseline characteristics	Maintenance dose 1	Maintenance dose 2	Total
Number of patients, *n*^a^	31	9	41
FVIII inhibitor status, *n* (%)			
Negative	28 (90.3)	8 (88.9)	37 (90.2)
Positive	3 (9.7)	1 (11.1)	4 (9.8)
Weight group, *n* (%)			
30–<60 kg	6 (19.4)	1 (11.1)	6 (14.6)
≥60 kg	25 (80.6)	8 (88.9)	35 (85.4)
Weight in kg, mean ± SD	74.2 ± 14.2	75.9 ± 11.3	75.3 ± 13.1
Age group, *n* (%)			
12-17 y	4 (12.9)	3 (33.3)	6 (14.6)
18-64 y	27 (87.1)	6 (66.7)	35 (85.4)
Age (y), mean ± SD	32.7 ± 12.3	30.3 ± 15.5	33.1 ± 12.6
Race, *n* (%)			
White	25 (80.6)	6 (66.7)	33 (80.5)
Asian	1 (3.2)	1 (11.1)	2 (4.9)
Black or African American	4 (12.9)	1 (11.1)	4 (9.8)
Other	1 (3.2)	1 (11.1)	2 (4.9)

FVIII, factor VIII; MAD, multiple ascending doses.

a Patients may be counted more than once because they were allowed to switch between extension starting doses and maintenance doses.

During the MAD extension phase, 2 patients discontinued treatment: 1 patient owing to a TEAE of Hodgkin lymphoma (in extension starting dose 3) and another owing to a patient decision after a sequence of traumatic and spontaneous joint bleeds (in extension maintenance dose 1 [QW]). Thirty-nine patients completed the MAD extension phase.

### 3.2 Individual treatment duration and switches

**Figure 1:**
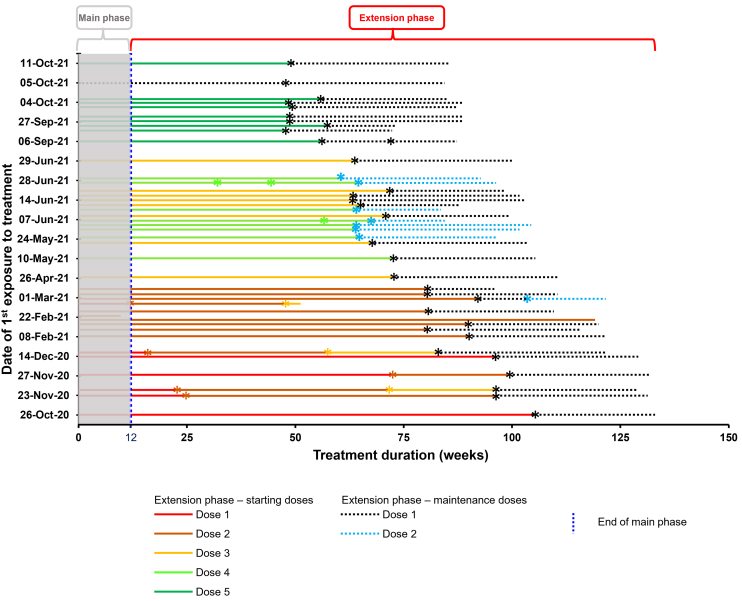
Individual treatment duration and switches during the FRONTIER1 MAD part main and extension phases Each horizontal line represents an individual patient. Gray shading indicates the main phase (week 0-week 12). ^a^Time points at which patients switched from one dose to another dose. Patients could switch between extension starting doses prior to receiving the extension maintenance dose. Each extension starting dose contained 2 doses, depending on body weight; there was a small number of patients who changed dose within an extension starting dose, which was permitted by the protocol. Patients enrolled in the FRONTIER1 MAD extension study for ≥12 weeks were allowed to transfer to the long-term study FRONTIER4 (NCT05685238). MAD, multiple ascending doses; Q4W, once every 4 weeks; QW, once every week

The total Mim8 exposure time during the MAD extension was 69.5 patient-years, comprising 46.4 years of extension starting dose and 23.2 years of extension maintenance doses. The number of patients exposed to Mim8 extension starting doses in cohorts 1, 2, 3, 4, and 5 during the initial MAD extension phase were 6, 13, 11, 8, and 10 patients, respectively (patients may be counted more than once since they were allowed to switch between extension starting doses). Subsequently, 39 patients received extension maintenance doses during the MAD extension phase according to body weight (30–<60 kg or ≥60 kg): 31 patients received 6 or 11 mg of Mim8 QW, respectively, and 9 patients received 30 or 46 mg of Mim8 Q4W, respectively. The latter dosing includes 1 patient who switched from QW maintenance dosing to Q4W maintenance dosing during the extension. One patient opted to remain on extension starting dose 2. Detailed information on individual treatment durations and switches is illustrated in Figure 1.

### 3.3 Safety

During the extension, 35 patients (85.4%) experienced 174 TEAEs, mostly mild or moderate, signifying a rate of 2.5 TEAEs per patient per year of exposure (Table 2). Thirteen (31.7%) patients experienced 17 TEAEs that were judged to be possibly or probably related to Mim8 (Supplementary Table 2). Rates of TEAEs were similar across the different extension starting doses and maintenance doses. There was no dose dependence of TEAE rate, type, causality, or severity.

**Table 2:** Treatment-emergent adverse events during the multiple ascending doses part extension phase doses.

Safety parameters	Starting dose 1		Starting dose 2		Starting dose 3		Starting dose 4		Starting dose 5		Maintenance dose 1		Maintenance dose 2		Total	
	*n* (%)	E (R)	*n* (%)	E (R)	*n* (%)	E (R)	*n* (%)	E (R)	*n* (%)	E (R)	*n* (%)	E (R)	*n* (%)	E (R)	*N* (%)	E (R)
NUMBER of patients, *n*^a^	6		13		11		8		10		31		9		41	
Total exposure time (y)	5.20		16.05		9.48		8.21		7.42		18.16		4.99		69.5 (range: 0.75-2.29)	
Adverse events	3 (50.0)	6 (1.2)	11 (84.6)	36 (2.2)	7 (63.6)	31 (3.3)	6 (75.0)	19 (2.3)	7 (70.0)	16 (2.2)	17 (54.8)	56 (3.1)	5 (55.6)	10 (2.0)	35 (85.4)	174 (2.5)
Serious adverse events	–	–	1 (7.7)	1 (0.1)	2 (18.2)	2 (0.2)	1 (12.5)	2 (0.2)	–	–	2 (6.5)	2 (0.1)	–	–	5 (12.2)	7 (0.1)
Severity																
Severe	–	–	–	–	1 (9.1)	1 (0.1)	1 (12.5)	1 (0.1)	–	–	–	–	–	–	2 (4.9)	2 (0.0)
Moderate	1 (16.7)	2 (0.4)	6 (46.2)	13 (0.8)	5 (45.5)	6 (0.6)	2 (25.0)	2 (0.2)	2 (20.0)	3 (0.4)	4 (12.9)	8 (0.4)	1 (11.1)	1 (0.2)	17 (41.5)	35 (0.5)
Mild	3 (50.0)	4 (0.8)	9 (69.2)	23 (1.4)	6 (54.5)	24 (2.5)	6 (75.0)	16 (1.9)	7 (70.0)	13 (1.8)	15 (48.4)	48 (2.6)	5 (55.6)	9 (1.8)	33 (80.5)	137 (2.0)
Probable/possible Mim8-related TEAEs	1 (16.7)	2 (0.4)	4 (30.0)	6 (0.4)	2 (18.2)	2 (0.2)	1 (12.5)	1 (0.1)	1 (10.0)	1 (0.1)	5 (16.1)	5 (0.3)	–	–	13 (31.7)	17 (0.2)
No. of injections, *n*	267		832		493		102		385		975		74		3128	
Injection-site reactions	1 (16.7)	1 (0.2)	2 (15.4)	2 (0.1)	2 (18.2)	2 (0.2)	–	–	–	–	1 (3.2)	1 (0.1)	–	–	6 (14.6)	6 (0.1)

%, percentage of patients with adverse event; E, number of adverse events; MAD, multiple ascending doses; *n*, number of patients with adverse event; R, number of adverse events per patient-year of exposure (E/total time in trial); TEAE, treatment-emergent adverse event.

a Patients may be counted more than once because they were allowed to switch between dosing cohorts.

Five patients (12.2%) had 7 serious TEAEs, comprised single events of headache and urticaria (both mild); febrile neutropenia, Hodgkin disease, and craniocerebral injury (all moderate); and seizure and traumatic intracranial hemorrhage (both severe). All serious TEAEs were judged to be unlikely related to Mim8, and all patients were recovered or recovering.

One mild, nonserious reaction with the (Medical Dictionary for Regulatory Activities-preferred term) urticaria (possibly related to Mim8), under the hypersensitivity reactions category, was reported in extension starting dose 2. The patient recovered after 2 days without requiring treatment for the TEAE or a change in Mim8 dosing.

Six patients (14.6%) experienced 6 injection-site reactions, representing 0.2% of the 3128 injections administered throughout the MAD extension phase (Table 2). Five of these cases were mild, and 1 was moderate; all cases were resolved (Supplementary Table 3). There was 1 patient withdrawal due to a TEAE (Hodgkin lymphoma, unlikely related to Mim8). There was no occurrence of anti–Mim8 antibodies.

### 3.4 Additional safety endpoints

**Figure 2:**
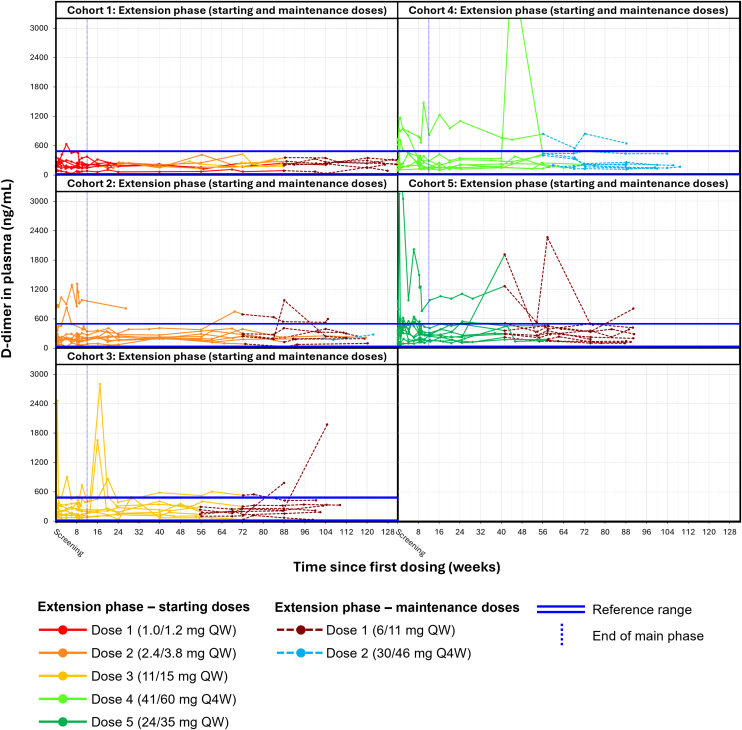
Individual profiles of D-dimer during the FRONTIER1 MAD part main and extension phases. D-dimer levels during the main and extension phases of the MAD part of FRONTIER1. D-dimer level outside the displayed range in cohort dose 4 was 4295 ng/mL (1 participant). MAD, multiple ascending doses; Q4W, once every 4 weeks; QW, once every week.

No dose-dependent changes in D-dimer levels were observed during the MAD main and extension phases. D-dimer values mostly remained within the normal range of < 500 ng/mL [15] and were stable over time (Figure 2).

**Figure 3:**
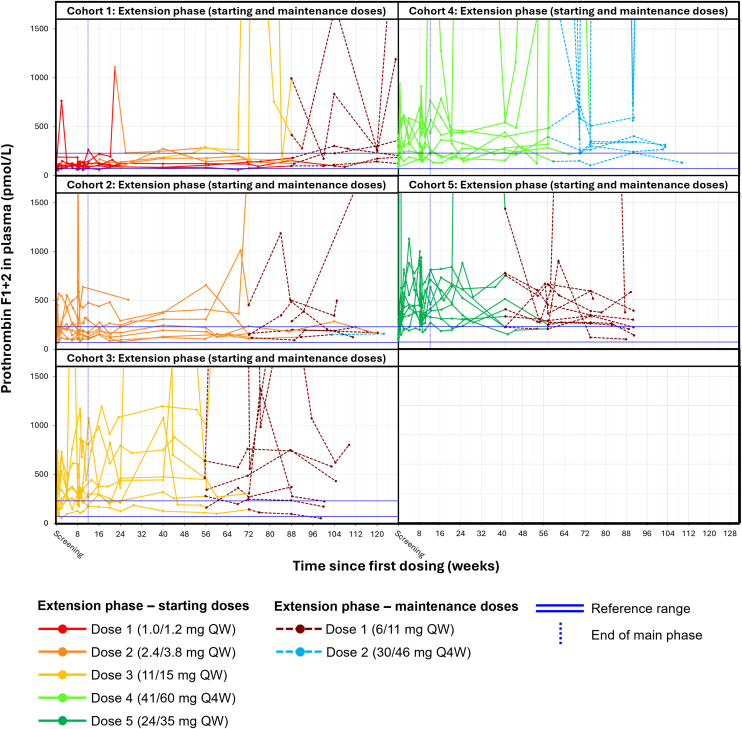
Individual profiles of prothrombin fragment 1 + 2 during the FRONTIER1 MAD part main and extension phases. Prothrombin fragment 1 + 2 levels during the main and extension phases of the MAD part of FRONTIER1. Prothrombin levels outside the displayed scale ranged between 1731 and 12 936 pmol/L (2 participants in cohort dose 1), 1991 and 2110 pmol/L (3 participants in cohort dose 2), 2534 and 19 388 pmol/L (3 participants in cohort dose 3), 2206 and 24 000 pmol/L (4 participants in cohort dose 4), and 9791 and 24 000 pmol/L (2 participants in cohort dose 5). MAD, multiple ascending doses; Q4W, once every 4 weeks; QW, once every week.

Prothrombin fragment 1 + 2 showed more variability than D-dimer, with a slight dose-dependent increase during the main phase (from baseline to week 12) (Figure 3). Individual profiles of prothrombin fragment 1 + 2 stabilized after patients achieved steady-state concentrations of Mim8.

Most outliers were observed in samples from a single site. There appeared to be no correlation between increases in prothrombin fragment 1 + 2 and changes in the levels of D-dimer or fibrinogen based on graphs using the same time scale.

### 3.5 Pharmacokinetics

**Figure 4:**
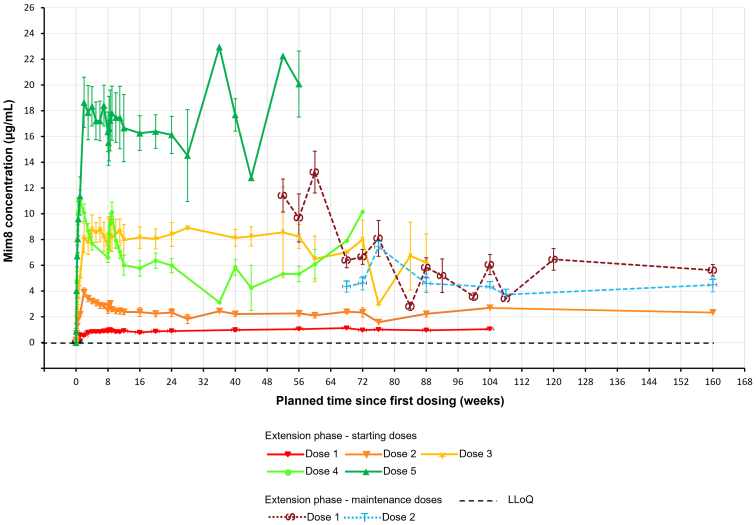
Mean profiles of Mim8 concentration during the FRONTIER1 MAD part main and extension phase. Data are shown as mean ± SD. Pre-dose measurements below LLoQ values are set to 0. Vertical lines indicate pharmacokinetics session 2. Confidence interval bars are only applicable when data from multiple patients are available for the same timepoint. All patients enrolled in the MAD extension phase had an end-of-treatment measurement taken at visit 43 (week 160). Patients who transferred to FRONTIER4 (NCT05685238) had an end-of-treatment measurement taken at visit 43, presented as data at week 160. LLoQ, lower limit of quantification; MAD, multiple ascending doses.

Individual profiles of Mim8 concentration (Supplementary Figure 2) and mean PK profiles (Figure 4) were consistent with Mim8 doses across all extension starting doses and maintenance doses. Mim8 exposure stabilized within a 4.3 to 7.1 μg/mL range during extension maintenance dosing. Mean Mim8 concentrations for the maintenance QW (maintenance 1) and Q4W (maintenance 2) dose groups were comparable (Figure 4).

### 3.6 Pharmacodynamics

**Figure 5:**
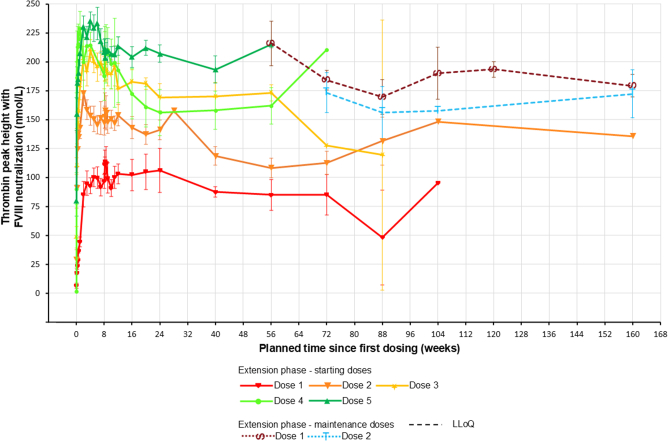
Mean profiles of thrombin peak height with FVIII neutralization during the FRONTIER1 MAD part main and extension phase. Data are shown as mean ± SD. Day 1 pre-dose data are not included in the plot owing to limited amount of data. Vertical lines indicate pharmacokinetics session 2. Confidence interval bars are only applicable when data from multiple patients are available for the same timepoint. All patients enrolled in the MAD extension phase had an end-of-treatment measurement taken at visit 43 (week 160). Patients who transferred to FRONTIER4 (NCT05685238) had an end-of-treatment measurement taken at visit 43, presented as data at week 160. MAD, multiple ascending doses.

Individual profiles of thrombin peak height with FVIII neutralization were consistent with Mim8 doses across extension starting doses and maintenance 1 (QW) and maintenance 2 (Q4W) doses. During extension maintenance dosing, they remained stable and within the target range of 150 to 200 nM (Supplementary Figure 3). Mean peak thrombin levels remained stable in the MAD extension phase (Figure 5).

### 3.7 Treated bleeding episodes

Over 69.5 patient-years of Mim8 exposure during the MAD extension, 115 bleeds were treated with factor products (FVIII or recombinant activated FVII) and were categorized as spontaneous (*n* = 66, 57.4%), traumatic (*n* = 46, 40.0%), or surgical (*n* = 3, 2.6%) (Table 3). The percentage of patients with zero bleeds during QW maintenance dosing (total exposure time, 18.16 years) was 67.7% (*n* = 21/31). The percentage of patients with zero bleeds during Q4W maintenance dosing (total exposure time, 4.99 years) was 88.9% (*n* = 8/9). Most bleeds (*n* = 112/115; 97.0%) were mild/moderate in severity. Three severe bleeds were observed in 3% of patients (*n* = 2/41): 1 patient had 1 traumatic bleed in the central nervous system, and another patient had 2 spontaneous bleeds in the right ankle (treated with FVIII).

**Table 3:** Treated bleeding episodes during the FRONTIER1 multiple ascending doses part extension phase.

Bleeding outcomes	Starting dose 1	Starting dose 2	Starting dose 3	Starting dose 4	Starting dose 5	Maintenance dose 1	Maintenance dose 2	Total
No. of patients, *n*^a^	6	13	11	8	10	31	9	41
Mean exposure time (wks)	45.2	64.4	44.9	35.7	38.7	29.6	28.9	
Total exposure time (y)	5.2	16.0	9.5	8.2	7.4	18.2	5.0	69.5
Number of bleeds	19	19	11	3	36^b^	26^b^	1	115
No. of patients with bleeds, *n* (%)	6 (100)	7 (53.8)	7 (63.6)	3 (37.5)	4 (40.0)	10 (32.3)	1 (11.1)	25 (61.0)
No. of patients with zero bleeds, *n* (%)	–	6 (46.2)	4 (36.4)	5 (62.5)	6 (60.0)	21 (67.7)	8 (88.9)	16 (39.0)
No. of severe bleeds	–	–	1	–	–	2	–	3
No. of patients with severe bleeds, *n* (%)	–	–	1 (9.1)	–	–	1 (3.2)	–	2 (4.9)
Cause of bleed, *n* (%)								
Spontaneous	10 (52.6)	5 (26.3)	2 (18.2)	–	34 (94.4)	14 (53.8)	1 (100)	66 (57.4)
Traumatic	9 (47.4)	12 (63.2)	8 (72.7)	3 (100)	2 (5.6)	12 (46.2)	–	46 (40.0)
Surgical	–	2 (10.5)	1 (9.1)	–	–	–	–	3 (2.6)
Site of bleed, *n* (%)								
Joint	9 (47.4)	10 (52.6)	3 (27.3)	2 (66.7)	35 (97.2)	22 (84.6)	1 (100)	82 (71.3)
Spontaneous	3 (30.0)	3 (30.0)	1 (33.3)	–	34 (97.1)	14 (63.6)	1 (100)	56 (68.3)
Traumatic	6 (60.0)	7 (70.0)	2 (66.7)	2 (100)	1 (2.9)	8 (36.4)	–	26 (31.7)
Nonjoint	10 (52.6)	9 (47.4)	8 (72.7)	1 (33.3)	1 (2.8)	4 (15.4)	–	33 (28.7)

MAD, multiple ascending doses.

a Patients may be counted more than once because they were allowed to switch between dosing cohorts.

b A single patient accounted for 31 bleeds in extension starting dose 5, and a further 6 bleeds in maintenance dose 1.

During the MAD extension phase, a single patient in extension starting dose cohort 5 accounted for 32% of bleeds in the study period. The patient reported 31 bleeds in 251 days of the starting dose, and a further 6 bleeds in 170 days on weekly extension maintenance 1 (QW) dosing (all bleeds were in the joints). There was no evidence of any antidrug antibodies. Of note, this patient had previously experienced 58 bleeds despite prophylactic FVIII treatment administered once every 3 days in the 12 months before entry to FRONTIER1 MAD part main phase and a further 8 bleeds during the FRONTIER1 MAD part main phase.

## 4 Discussion

The FRONTIER1 MAD part extension phase assessed the long-term safety, PK and PD of subcutaneous Mim8. Adult and adolescent people with hemophilia A with or without FVIII inhibitors had a total Mim8 exposure of 69.5 patient-years.

During the MAD extension phase, no safety concerns were found with Mim8. Injection-site reaction rates were relatively low. Most TEAEs were mild or moderate, unlikely related to Mim8, and resolved without dose changes. No thromboembolic events occurred, and no Mim8 antidrug antibodies were observed.

The safety outcomes of Mim8 were consistent with results from the FRONTIER1 MAD part main phase [13]. There were relatively low rates of injection-site reactions in both the MAD extension phase, similar to the main phase, in which 4 injection-site reactions were observed in 418 injections (1.0%), affecting 4 of 42 participants (9.5%) [13]. The proportion of patients experiencing injection-site reactions with Mim8 was comparable or lower than other subcutaneous agents that are dosed QW (marstacimab: 6.1%–10.8%), or QW to once every month (QM) (emicizumab: 20.8%–35.2%) [16,17]. The rate of injection-site reactions per patient-year of exposure for Mim8 (0.1) was also lower than that observed for the daily concizumab regimen (0.4) [18].

Furthermore, in line with the results of the MAD part main phase, treatment with Mim8 did not induce any detectable neutralizing anti–Mim8 antibodies [13]. In contrast, neutralizing antidrug antibodies have been reported with other agents, including marstacimab in up to 6 of 116 patients (5%, phase 3 study, 12-month follow-up), concizumab in 12 of 185 patients (6.5%, pooled clinical trials data, ≥72-week follow-up), and emicizumab in 3 of 401 patients (0.7%, pooled phase 3 trials data, ≥144-week follow-up) [17,19,20]. Because these figures come from separate trials with different dosing schedules and follow-up times, the cross-compound comparisons are descriptive only and should not be interpreted as head-to-head evidence. Individual PK profiles of Mim8 concentration remained consistent across extension starting and maintenance doses. Comparable steady-state Mim8 exposure was observed with QW and Q4W dosing frequencies, supporting the feasibility for both tiered dosing regimens to accommodate patients with different needs. This consistency in Mim8 exposure was also reflected in the observed thrombin generation profiles. Mim8 was rationally engineered to increase FIXa-cofactor activity and more closely mimic native FVIII function [11]. *In vitro*, its anti-FIXa arm enhances FIXa proteolytic activity by >20 000-fold over natural FIXa alone [11]. In the FRONTIER1 MAD main phase, Mim8 achieved comparable peak thrombin at ∼15-fold lower plasma concentrations than emicizumab, demonstrating its higher potency [12]. However, pharmacologic potency does not necessarily translate into clinical haemostatic benefit, and the clinical relevance of these findings remains to be confirmed. Thrombin generation remained stable in the present extension after patients switched to maintenance doses, and phase 3 studies of the FRONTIER program will evaluate how this PD profile translates into clinical efficacy.

D-dimer values were assessed as potential indicators of thromboembolic risk. Similar to the FRONTIER1 MAD part main phase [13] and emicizumab data [21], no dose-dependent pattern of increase in D-dimer values was observed during the MAD extension phase. D-dimer values were mostly within the normal range and were stable over time. The potential importance of D-dimer values is unknown and requires further clinical investigation.

As reported previously, there was a dose-dependent increase in prothrombin fragment 1 + 2 values during the MAD part main phase [13]; this dose dependence stabilized at the level of extension starting dose 3. Overall, there was more variability observed in prothrombin fragment 1 + 2 levels compared with D-dimer; this was expected [22], given the high sensitivity of this assay to variations in sampling, handling, and storage methods [23]. Most outliers were observed in samples collected at a single site, suggesting that this level of variability could have been due to a sampling issue. Importantly, none of the outlier values were in correlation with any TEAEs and therefore judged by the investigators as clinically nonsignificant. Individual PK concentrations were within the expected range for each extension starting dose and for the 2 extension maintenance doses. Thrombin peak height levels remained stable following dose switches. Taken together, the stability of these parameters over time reinforces the pharmacodynamic predictability of Mim8 during long-term treatment.

In addition to stable thrombin generation across maintenance groups, 67.7% of patients on QW dosing and 88.9% of patients on Q4W dosing experienced zero treated bleeds. These data suggest that both regimens were associated with meaningful bleed protection over time and align with outcomes seen in studies of emicizumab, which reported a median of 67% of patients on prophylaxis achieving zero bleeds across 15 clinical trials and real-world cohorts [24]. While the study was not powered to statistically compare the 2 dose frequencies, the high proportion of patients with zero bleeds and the absence of dose-related safety signals support the continued investigation of both regimens in phase 3.

Bleeding outcomes were skewed by 1 patient who accounted for 31 of 36 bleeds in extension starting dose 5 and a further 6 of 26 bleeds in the extension QW maintenance dosing. The number of required treatments decreased from 31 in 251 days to 6 in 170 days after the patient transitioned to extension QW maintenance dosing. This patient had a history of a large number of bleeds before enrolling in FRONTIER1 (despite prophylactic factor treatment) and during the MAD part main phase of the trial. Subsequently, the patient enrolled in the long-term, open-label, ongoing FRONTIER4 study. The patient’s high bleed count may partly reflect inter-individual differences in bleeding phenotype, an effect that has been reported even among patients regardless of hemophilia severity [25,26].

This trial was a long-term assessment, which allowed for monitoring potential delayed or cumulative effects of Mim8. Additionally, comprehensive data were collected on multiple drug doses, frequencies, and weight ranges.

Potential limitations of the study include a relatively small patient population and a shorter duration of time to assess extension maintenance doses of Mim8. Phase 3 studies have been conducted to further investigate safety outcomes with Mim8 in people with hemophilia A with or without inhibitors (FRONTIER2 [NCT05053139] and FRONTIER3 [NCT05306418]). These phase 3 studies will evaluate Mim8 using a tiered dosing approach and pre-filled pen device designed to reduce the burden of administration. In addition, a long-term, open-label safety and efficacy trial (FRONTIER4 [NCT05685238]) is planned to include people with hemophilia A, with or without inhibitors, who have completed participation in previous FRONTIER trials. FRONTIER4 will study once-every-2-weeks dosing, in addition to QW and QM dosing regimens.

## 5 Conclusion

During the FRONTIER1 MAD part extension study, no safety concerns were observed during treatment with Mim8 in adult and adolescent people with severe hemophilia A with or without inhibitors. A low rate of TEAEs and injection-site reactions was observed, and there were no thromboembolic events or occurrences of Mim8 antidrug antibodies. The safety outcomes of Mim8 were consistent with results from the main phase of the MAD part of FRONTIER1. Mim8 exposure and thrombin generation were dose-dependent, and the majority of patients had no bleeds during extension maintenance dosing. These data suggest that Mim8 is well tolerated and support weekly to once-every-4-weeks maintenance dosing. Data from the extension study support further clinical development of Mim8 in phase 3 studies.
