# Supplementary material for: FRONTIER1 multiple ascending dose extension: a safety, tolerability, pharmacokinetics, and pharmacodynamics study of Mim8 in people with hemophilia A
Source: Res Pract Thromb Haemost. 2025 Oct 8;9(7):103207. doi: 10.1016/j.rpth.2025.103207 (PMC12617623; doi:10.1016/j.rpth.2025.103207)
Supplement: Supplementary Material [file mmc1.docx]

# Supplementary Materials

# Title page

**FRONTIER1 multiple ascending dose extension: a safety, tolerability, pharmacokinetics, and pharmacodynamics study of Mim8 in patients with haemophilia A**

Pratima Chowdary,^1^* Steven R Lentz,^2^ Lidia Gil,^3^ Francisco J López-Jaime,^4^ Jerzy Windyga,^5^ Wan Hui Ong Clausen,^6^ Peter Nørkjær Laursen,^6^ Johnny Mahlangu^7^

1. Katharine Dormandy Haemophilia and Thrombosis Centre, Royal Free Hospital, Department of Haematology, University College London, London, UK
2. Department of Internal Medicine, University of Iowa, Iowa City, IA, USA
3. Department of Hematology and Bone Marrow Transplantation, Poznań University of Medical Sciences, Poznań, Poland
4. Department of Hemostasis and Thrombosis, Hematology Service, Hospital Universitario Regional de Málaga, Málaga, Spain
5. Department of Hemostasis Disorders and Internal Medicine, Laboratory of Hemostasis and Metabolic Diseases, Institute of Hematology and Transfusion Medicine, Warsaw, Poland
6. Novo Nordisk A/S, Søborg, Denmark
7. Department of Molecular Medicine and Haematology, University of the Witwatersrand, National Health Laboratory Service, and Charlotte Maxeke Johannesburg Academic Hospital, Johannesburg, South Africa

## *Corresponding author:

Pratima Chowdary

Katharine Dormandy Haemophilia and Thrombosis Centre, Royal Free Hospital, London, NW3 2QG, UK

Tel: 0044 20 7472 6835

Email: p.chowdary@ucl.ac.uk

## Tables

Supplementary Table 1 Dosing regimens in the FRONTIER1 MAD part extension phase

|  | **Extension phase  starting doses** | | | | | **Extension phase  maintenance doses** | |
| --- | --- | --- | --- | --- | --- | --- | --- |
|  | **Starting dose 1** | **Starting  dose 2** | **Starting  dose 3** | **Starting  dose 4** | **Starting  dose 5** | **Maintenance**  **dose 1** | **Maintenance  dose 2** |
| Frequency | QW | QW | QW | Q4W | QW | QW | Q4W |
| Mim8 dosing (mg) |  |  |  |  |  |  |  |
| Body weight 30–<60 kg | 1 | 2.4 | 11 | 41 | 24 | 6 | 30 |
| Body weight ≥60 kg | 1.2 | 3.8 | 15 | 60 | 35 | 11 | 46 |

MAD, multiple ascending doses; Q4W, once every four weeks; QW, once weekly.

**Supplementary Table** 2 Total probable/possible Mim8-related TEAEs during the FRONTIER1 MAD part extension phase

|  | **Number of patients with TEAE, *n* (%)** | **Number of TEAEs, *n*** | **Rate^a^** |
| --- | --- | --- | --- |
| Probable/possible Mim8-related TEAEs | **13 (31.7)** | **17** | **0.2** |
| Probable/possible Mim8-related TEAEs by System Organ Class |  |  |  |
| General disorders and administration-site conditions | **6 (14.6)** | **7** | **0.1** |
| Injection-site erythema | 1 (2.4) | 1 | 0 |
| Injection-site haematoma | 2 (4.9) | 2 | 0 |
| Injection-site haemorrhage | 2 (4.9) | 2 | 0 |
| Injection-site induration | 1 (2.4) | 1 | 0 |
| Injection-site pain | 1 (2.4) | 1 | 0 |
| Hepatobiliary disorders | **1 (2.4)** | **1** | **0** |
| Gallbladder polyp | 1 (2.4) | 1 | 0 |
| Injury, poisoning and procedural complications | **3 (7.3)** | **3** | **0** |
| Incorrect dose administered | 2 (4.9) | 2 | 0 |
| Incorrect route of product administration | 1 (2.4) | 1 | 0 |
| Investigations | **1 (2.4)** | **1** | **0** |
| Prothrombin fragment 1 + 2 increased | 1 (2.4) | 1 | 0 |
| Musculoskeletal and connective tissue disorders | **2 (4.9)** | **2** | **0** |
| Arthralgia | 2 (4.9) | 2 | 0 |
| Renal and urinary disorders | **1 (2.4)** | **1** | **0** |
| Proteinuria | 1 (2.4) | 1 | 0 |
| Respiratory, thoracic and mediastinal disorders | **1 (2.4)** | **1** | **0** |
| Epistaxis | 1 (2.4) | 1 | 0 |
| Skin and subcutaneous tissue disorders | **1 (2.4)** | **1** | **0** |
| Urticaria | 1 (2.4) | 1 | 0 |

^a^Number of TEAEs per subject-year of exposure (number of TEAEs/total time in trial).

*n*, number of patients; MAD, multiple ascending doses; TEAE, treatment-emergent adverse event.

Supplementary Table 3 Detailed injection-site reactions data for FRONTIER1 MAD part extension phase

| **Cohort** | **Adverse event^a^** | **Duration** | **Adverse event severity** | **Outcome** |
| --- | --- | --- | --- | --- |
| Starting dose 1 | Injection-site haematoma | 1 d, 15 h, 45 min | Moderate | Recovered/ resolved |
| Starting dose 1 | Injection-site haematoma | 4 d, 23 h, 59 min | Mild | Recovered/ resolved |
| Starting dose 1 | Injection-site pain | 3 min | Mild | Recovered/ resolved |
| Starting dose 2 | Injection-site haemorrhage | 2 d, 16 h, 59 min | Mild | Recovered/ resolved |
| Starting dose 3 | Injection-site haemorrhage | 4 d, 0 h, 0 min | Mild | Recovered/ resolved |
| Starting dose 3 | Injection-site erythema | 2 d | Mild | Recovered/ resolved |

^a^MedDRA-Preferred Term.

d, day; h, hour; min, minute; MAD, multiple ascending doses; MedDRA, Medical Dictionary for Regulatory Activities.

Supplementary Figure 1 Study design of the FRONTIER1 MAD part: main phase and extension phases


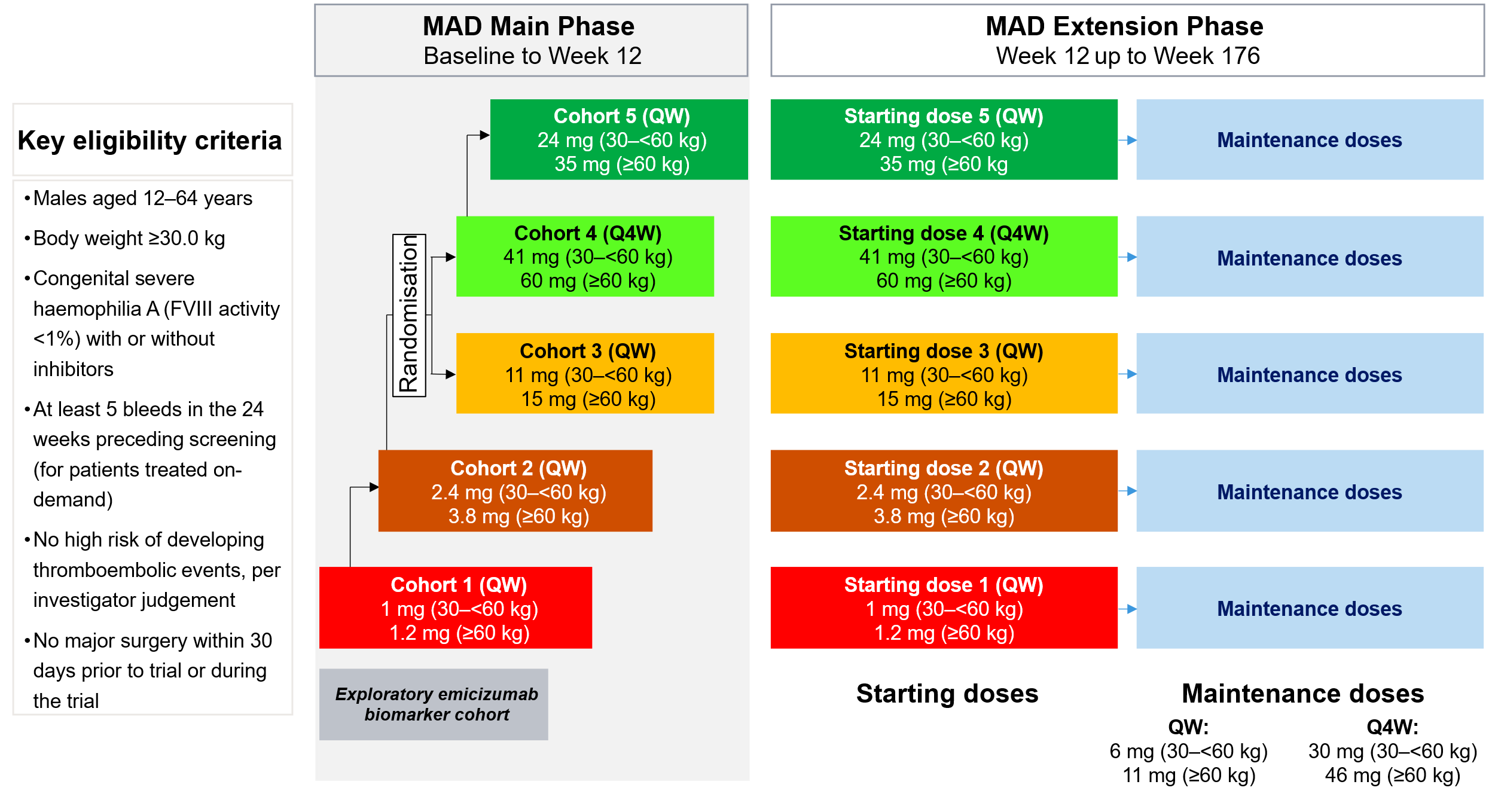


Colours denote cohort numbering; blue boxes denote maintenance dosing. Black arrows in the MAD main phase indicate ascending Mim8 doses. FVIII, factor VIII; MAD, multiple ascending dose; QW, once weekly; Q4W, once every four weeks.

**Supplementary Figure 2** Individual profiles of Mim8 concentration during the FRONTIER1 MAD part main and extension phases


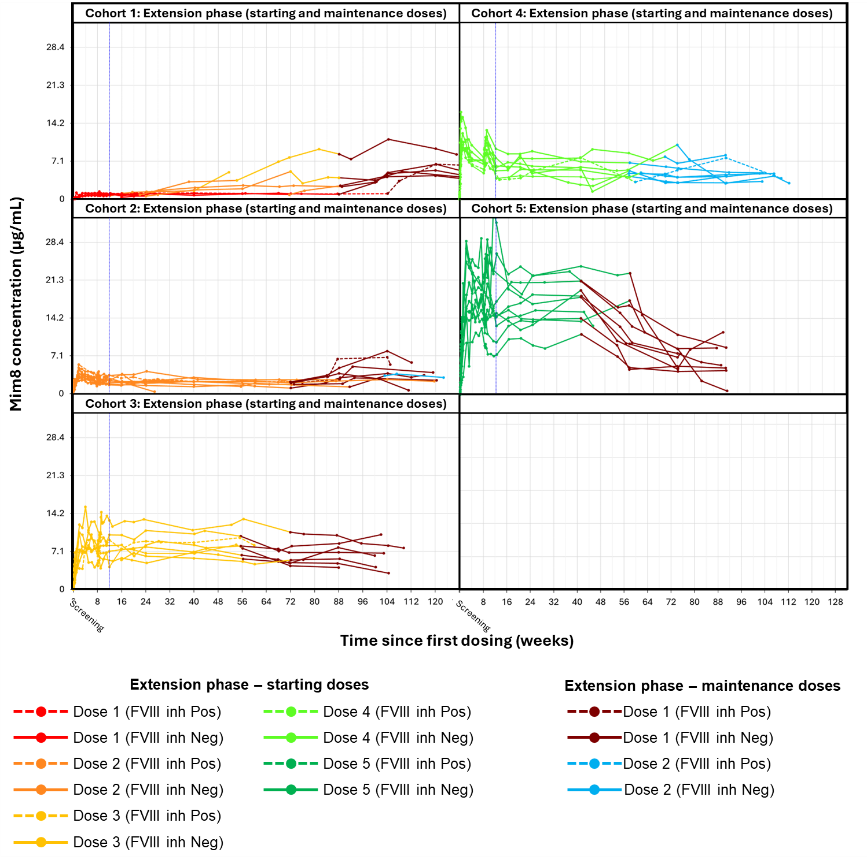


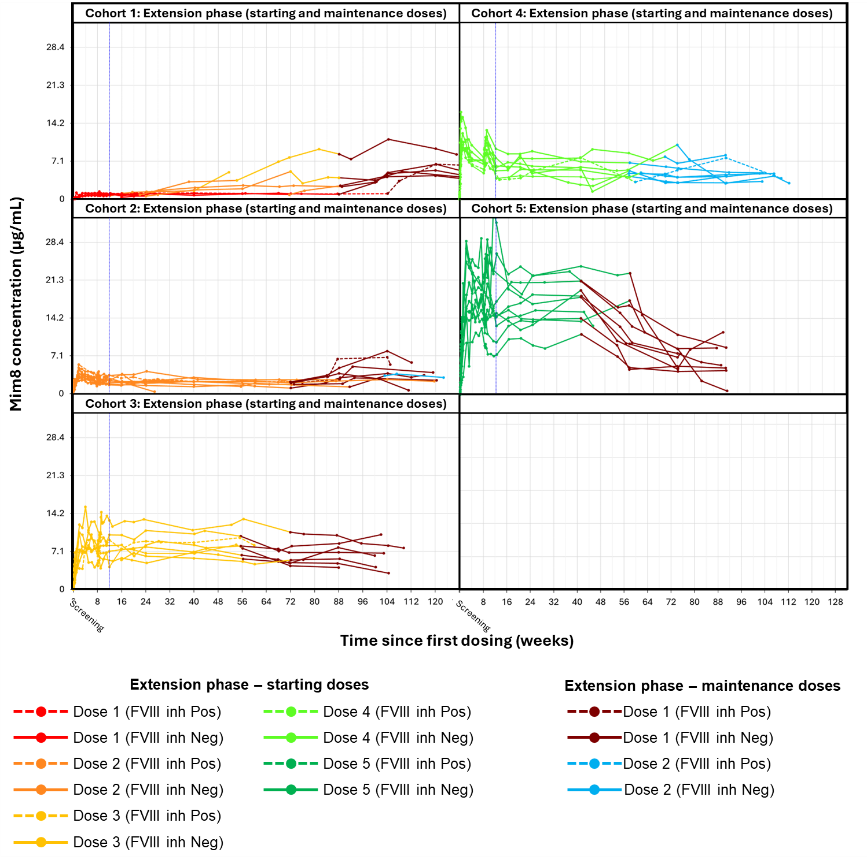


Concentration of Mim8 in patients with FVIII inhibitors and without FVIII inhibitors. Pre-dose measurements below LLoQ are set to 0.

FVIII, factor VIII; inh, inhibitor; LLoQ, lower limit of quantification; MAD, multiple ascending doses; Neg, FVIII inhibitor negative; Pos, FVIII inhibitor positive; Q4W, once every four weeks; QW, once every week.

Supplementary Figure 3 Individual profiles of thrombin peak height with FVIII neutralisation during the FRONTIER1 MAD part main and extension phases


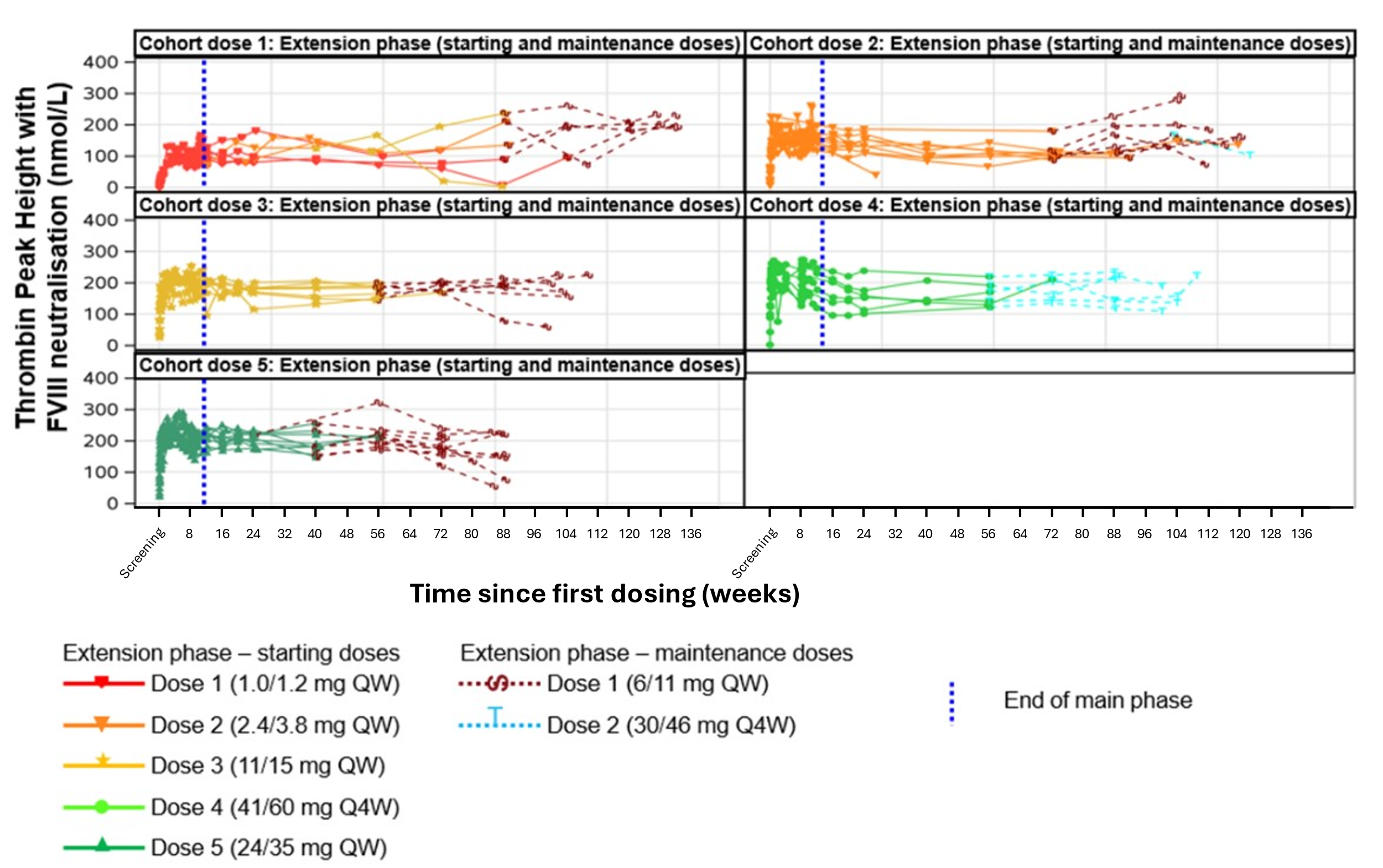


Individual measurements of thrombin peak height with FVIII neutralisation. One subject discontinued treatment and had a follow-up of 119 days after last dose.

FVIII, factor VIII, MAD, multiple ascending dose; Q4W, once every four weeks; QW, once every week.
